# Supplementary material for: Demographic and genetic characterization of harvested Corbicula fluminea populations
Source: PeerJ. 2020 Sep 4;8:e9657. doi: 10.7717/peerj.9657 (PMC7476495; doi:10.7717/peerj.9657)
Supplement: File S1 — DNA sequence data and the number of individuals and weight of Corbicula fluminea among the different habitats, sampling sites and seasons in the Poyang Lake Basin. These DNA sequence data, the number of individuals and weight were used for statistical analysis. [file peerj-08-9657-s001.docx]

**Supplemental Files**

DNA sequence data that support the findings of this study have been deposited in the National Center of Biotechnology Information (NCBI https://www.ncbi.nlm.nih.gov/). GenBank accession codes: DNA sequence data that support the findings of this study have been deposited in the National Center of Biotechnology Information with the primary GenBank accession code MN233672-MN233683.

>Seq1 *Corbicula fluminea* Hap1 mitochondria COI genome-MN233672

AACCTACCGGATCAAAAAATGAAGTGTAATTACGATCAGTTAACAACAGAGCCCCAGCTAACACAGGCATTGCAATCAACAAAATTCCAGTTACAACAATACATCAAAACATTGTCCGAATTAGCTTTTGCGCCCCAGGACGCATACAAAATCTGACAACAAAATTTGAAGCTAAAATTGAAGAAATACCCCCTAAATGAAGAGAAAAAACTAAATCTACTGACGGGCCAGAATGAGCAATATTTCAAAGAGGAGGATAAACAGTCCACCCAGTCCCAGCACCACTCTCAACATAAGCCGATCCTAACAAAAGCACTAGTAAAAGCCAAAATCTTAAATTATTTAATCGTGGAAAAGCTATATCAGGAGCCCTTAACATTAATGGAACAAGTCAATTTCCAAAACCACCCATCATTATTGGCATTACTAAAAAAAAAATTATTACTAAAGCATGAGCAGCAATATTATACAACTGCCCATCATCTAGTAAAGTCCCTGGAACAAGCTCCATTCGAATTACCCAAGCAGTTCCTATTAAACCAGATCAAAT

>Seq2 *Corbicula fluminea* Hap2 mitochondria COI genome-MN233673

AGCCTACCGGATCAAAAAATGAAGTGTAATTACGATCAGTTAATAACAGAGCCCCAGCTAATACAGGCATTGCAATCAACAAAATTCCAGTTACAACAATACATCAAAACATTGTCCGGATTAGTTTTTGAGCTCCAGGACGCATACAAAATCTGACAACAAAATTTGAAGCTAAAATTGAAGAAATACCCCCTAAATGAAGAGAGAAAACTAAATCTACTGACGGGCCAGAATGAGCAATATTTCAGAGAGGAGGATAAACAGTCCACCCAGTCCCAGCACCACTCTCAACATAAGCTGATCCCAACAAAAGCACTAGTAAAAGCCAAAATCTTAAATTATTTAACCGTGGAAAAGCTATATCAGGAGCCCTTAACATTAATGGAACAAGTCAATTTCCAAAACCACCCATTATTATTGGCATCACTAAAAAAAAAATTATTACTAAAGCATGAGCAGCAATATTATACAGCTGCCCATCATCTAATAAAGTCCCTGGAACAAGCTCCATTCGAACTACCCAAGCAGTTCCTATTAAACCAGATCAAAT

>Seq3 *Corbicula fluminea* Hap3 mitochondria COI genome-MN233674

AACCTACAGGATCAAAAAATGAAGTGTAATTACGATCAGTTAACAACAGAGCCCCAGCTAACACAGGCATTGCAATCAACAAAATTCCAGTTACAACAATACATCAAAACATTGTCCGAATTAGCTTTTGCGCTCCAGGACGCATACAAAATCTGACAACAAAATTTGAAGCTAAAATTGAAGAAATACCCCCTAAATGAAGAGAAAAAACTAAATCTACTGACGGGCCAGAATGAGCAATATTTCAAAGAGGAGGATAAACAGTCCACCCAGTCCCAGCACCACTCTCAACATAAGCCGATCCTAACAAAAGCACTAGTAAAAGCCAAAATCTTAAATTATTTAATCGTGGAAAAGCTATATCAGGAGCCCTTAACATTAATGGAACAAGTCAATTTCCAAAACCACCCATCATTATTGGCATTACTAAAAAAAAAATTATTACTAAAGCATGAGCAGCAATATTATACAACTGCCCATCATCTAGTAAAGTCCCTGGAACAAGCTCCATTCGAATTACCCAAGCAGTTCCTATTAAACCAGATCAAAT

>Seq4 *Corbicula fluminea* Hap4 mitochondria COI genome-MN233675

AACCTACCGGATCAAAAAATGAAGTGTAATTACGATCAGTTAACAACAGAGCCCCAGCTAACACAGGCATTGCAATCAACAAAATTCCAGTTACAACAATACATCAAAACATTGTCCGAATTAGCTTTTGCGCTCCAGGACGCATACAAAATCTGACAACAAAATTTGAAGCTAAAATTGAAGAAATACCCCCTAAATGAAGAGAAAAAACTAAATCTACTGACGGGCCAGAATGAGCAATATTTCAAAGAGGAGGATAAACAGTCCACCCAGTCCCAGCACCACTCTCAACATAAGCCGATCCTAACAAAAGCACTAGTAAAAGCCAAAATCTTAAATTATTTAATCGTGGAAAAGCTATATCAGGAGCCCTTAACATTAATGGAACAAGTCAATTTCCAAAACCACCCATCATTATTGGCATTACTAAAAAAAAAATTATTACTAAAGCATGAGCAGCAATATTATACAACTGCCCATCATCTAGTAAAGTCCCTGGAACAAGCTCCATTCGAATTACCCAAGCAGTTCCTATTAAACCAGATCAAAT

>Seq5 *Corbicula fluminea* Hap5 mitochondria COI genome-MN233676

AGCCTACCGGATCAAAAAATGAAGTGTAATTACGATCAGTTAATAACAGAGCCCCAGCTAATACAGGCATTGCAATCAACAAAATTCCAGTTACAACAATACATCAAAACATTGTCCGGATTAGTTTTTGAGCTCCAGGACGCATACAAAATCTGACAACAAAATTTGAAGCTAAAATTGAAGAAATACCCCCTAAATGAAGAGAGAAAACTAAATCTACTGACGGGCCAGAATGAGCAATATTTCAGAGAGGAGGATAAACAGTCCACCCAGTCCCAGCACCACTCTCAACATAAGCTGATCCCAACAAAAGCACTAGTAAAAGCCAAAATCTTAAATTATTTAACCGTGGAAAAGCTATATCAGGAGCCCTTAACATTAATGGAACAAGTCAATTTCCAAAACCACCCCTTATTATTGGCATCACTAAAAAAAAAATTATTACTAAAGCATGAGCAGCAATATTATACAGCTGCCCATCATCTAATAAAGTCCCTGGAACAAGCTCCATTCGAACTACCCAAGCAGTTCCTATTAAACCAGATCAAAT

>Seq6 *Corbicula fluminea* Hap6 mitochondria COI genome-MN233677

AGCCTACCGGATCAAAAAATGAAGTGTAATTACGATCAGTTAATAACAGAGCCCCAGCTAATACAGGCATTGCAATCAACAAAATTCCAGTTACAACAATACATCAAAACATTGTCCGGATTAGTTTTTGAGCTCCAGGACGCATACAAAATCTGACAACAAAATTTGAAGCTAAAATTGAAGAAATACCCCCTAAATGAAGAGAGAAAACTAAATCTACTGACGGGCCAGAATGAGCAATATTTTAGAGAGGAGGATAAACAGTCCACCCAGTCCCAGCACCACTCTCAACATAAGCTGATCCCAACAAAAGCACTAGTAAAAGCCAAAATCTTAAATTATTTAACCGTGGAAAAGCTATATCAGGAGCCCTTAACATTAATGGAACAAGTCAATTTCCAAAACCACCCATTATTATTGGCATCACTAAAAAAAAAATTATTACTAAAGCATGAGCAGCAATATTATACAGCTGCCCATCATCTAATAAAGTCCCTGGAACAAGCTCCATTCGAACTACCCAAGCAGTTCCTATTAAACCAGATCAAAT

>Seq7 *Corbicula fluminea* Hap7 mitochondria COI genome-MN233678

AACCTACCGGATCAAAAAATGAAGTGTAATTACGATCAGTTAACAACAGAGCCCCTGCTAACACAGGCATTGCAATCAACAAAATTCCAGTTACAACAATACATCAAAACATTGTCCGAATTAGCTTTTGCGCTCCAGGACGCATACAAAATCTGACAACAAAATTTGAAGCTAAAATTGAAGAAATACCCCCTAAATGAAGAGAAAAAACTAAATCTACTGACGGGCCAGAATGAGCAATATTTCAAAGAGGAGGATAAACAGTCCACCCAGTCCCAGCACCACTCTCAACATAAGCCGATCCTAACAAAAGCACTAGTAAAAGCCAAAATCTTAAATTATTTAATCGTGGAAAAGCTATATCAGGAGCCCTTAACATTAATGGAACAAGTCAATTTCCAAAACCACCCATCATTATTGGCATTACTAAAAAAAAAATTATTACTAAAGCATGAGCAGCAATATTATACAACTGCCCATCATCTAGTAAAGTCCCTGGAACAAGCTCCATTCGAATTACCCAAGCAGTTCCTATTAAACCAGATCAAAT

>Seq8 *Corbicula fluminea* Hap8 mitochondria COI genome-MN233679

AGCCTACCGGATCAAAAAATGAAGTGTAATTACGATCAGTTAATAACAGAGCCCCAGCTAATACAGGCATTGCAATCAACAAAATTCCAGTTACAACAATACATCAAAACATTGTCCGGATTAGTTTTTGAGCTCCAGGACGCATACAAAATTTGACAACAAAATTTGAAGCTAAAATTGAAGAAATACCCCCTAAATGAAGAGAGAAAACTAAATCTACTGACGGGCCAGAATGAGCAATATTTTAGAGAGGAGGATAAACAGTCCACCCAGTCCCAGCACCACTCTCAACATAAGCTGATTCCAACAAAAGCACTAGTAAAAGCCAAAATCTTAAATTATTTAACCGTGGAAAAGCTATATCAGGAGCCCTTAACATTAATGGAACAAGTCAATTTCCAAAACCACCCATTATTATTGGCATCACTAAAAAAAAAATTATTACTAAAGCATGAGCAGCAATATTATACAGCTGCCCATCATCTAATAAAGTCCCTGGAACAAGCTCCATTCGAACTACCCAAGCAGTTCCTTTTAACCCAGATCAAAT

>Seq9 *Corbicula fluminea* Hap9 mitochondria COI genome-MN233680

AACCTACCGGATCAAAAAATGAAGTGTAATTACGATCAGTTAACAACAGAGCCCCAGCTAACACAGGCATTGCAATCAACAAAATTCCAGTTACAACAATACATCAAAACATTGTCCGAATTAGCTTTTGCGCTCCAGGACGCATACAAAATCTGACAACAAAATTTGAAGCTAAAATTGAAGAAATACCCCCTAAATGAAGAGAAAAAACTAAATCTACTGACGGGCCAGAATGAGCAATATTTTAAAGAGGAGGATAAACAGTCCACCCAGTCCCAGCACCACTCTCAACATAAGCCGATCCTAACAAAAGCACTAGTAAAAGCCAAAATCTTAAATTATTTAATCGTGGAAAAGCTATATCAGGAGCCCTTAACATTAATGGAACAAGTCAATTTCCAAAACCACCCATCATTATTGGCATTACTAAAAAAAAAATTATTACTAAAGCATGAGCAGCAATATTATACAACTGCCCATCATCTAGTAAAGTCCCTGGAACAAGCTCCATTCGAATTACCCAAGCAGTTCCTATTAAACCAGATCAAAT

>Seq10 *Corbicula fluminea* Hap10 mitochondria COI genome-MN233681

AACCTACCGGATCAAAAAATGAAGTGTAATTACGATCAGTTAACAACAGAGCCCCAGCTAACACAGGCATTGCAATCAACAAAATTCCAGTTACAACAATACATCAAAACATTGTCCGAATTAGCTTTTGCGCTCCAGGACGCATACAAAATCTGACAACAAAATTTGAAGCTAAAATTGAAGAAATACCCCCTAAATGAAGAGAAAAAACTAAATCTACTGACGGGCCAGAATGAGCAATATTTCAAAGAGGAGGATAAACAGTCCACCCAGTCCCAACACCACTCTCAACATAAGCCGATCCTAACAAAAGCACTAGTAAAAGCCAAAATCTTAAATTATTTAATCGTGGAAAAGCTATATCAGGAGCCCTTAACATTAATGGAACAAGTCAATTTCCAAAACCACCCATCATTATTGGCATTACTAAAAAAAAAATTATTACTAAAGCATGAGCAGCAATATTATACAACTGCCCATCATCTAGTAAAGTCCCTGGAACAAGCTCCATTCGAATTACCCAAGCAGTTCCTATTAAACCAGATCAAAT

>Seq11 *Corbicula fluminea* Hap11 mitochondria COI genome-MN233682

AACCTACCGGATCAAAAAATGAAGTGTAATTACGATCAGTTAACAACAGAGCCCCAGCTAACACAGGCATTGCAATCAACAAAATTCCAGTTACAACAATACATCAAAACATTGTCCGAATTAGCTTTTGCGCTCCAGGACGCATACAAAATCTGACAACAAAATTTGAAGCTAAAATTGAAGAAATACCCCCTAAATGAAGAGAAAAAACTAAATCTACTGACGGGCCAGAATGAGCAATATTTCAAAGAGGAGGATAAACAGTCCACCCAGTCCCAGCACCACTCTCAACATAAGCCGATCCTAACAAAAGCACTAGTAAAAGCCAAAATCTTAAATTATTTAATCGTGGAAAAGCTATATCAGGAGCCCTTAACATTAATGGAACAAGTCAATTTCCAAAACCACCCATCATTATTGGCATTACTAAAAAAAAAATTATTACTAAAGCATGAGCAGCAATATTATACAACTGCCCATCATCTAGTAAAGTCCCTGGAACAAGCTGCATTCGAATTACCCAAGCAGTTCCTATTAAACCAGATCAAAT

>Seq12 *Corbicula fluminea* Hap12 mitochondria COI genome-MN233683

AACCTACCGGATCAAAAAATGAAGTGTAATTACGATCAGTTAACAACAGAGCCCCAGCTAACACAGGCATTGCAATCAACAAAATTCCAGTTACAACAATACATCAAAACATTGTCCGAATTAGCTTTTGCGCTCCAGGACGCATACAAAATCTGACAACAAAATTTGAAGCTAAAATCGAAGAAATACCCCCTAAATGAAGAGAAAAAACTAAATCTACTGACGGGCCAGAATGAGCAATATTTCAAAGAGGAGGATAAACAGTCCACCCAGTCCCAGCACCACTCTCAACATAAGCCGATCCTAACAAAAGCACTAGTAAAAGCCAAAATCTTAAATTATTTAATCGTGGAAAAGCTATATCAGGAGCCCTTAACATTAATGGAACAAGTCAATTTCCAAAACCACCCATCATTATTGGCATTACTAAAAAAAAAATTATTACTAAAGCATGAGCAGCAATATTATACAACTGCCCATCATCTAGTAAAGTCCCTGGAACAAGCTCCATTCGAATTACCCAAGCAGTTCCTATTAAACCAGATCAAAT
