# Supplementary material for: Demographic and genetic characterization of harvested Corbicula fluminea populations
Source: PeerJ. 2020 Sep 4;8:e9657. doi: 10.7717/peerj.9657 (PMC7476495; doi:10.7717/peerj.9657)
Supplement: File S2 [file peerj-08-9657-s002.docx]

**Supplemental Files 2**

Number of individual and weight of *Corbicula fluminea* in winter among the different habitats and sampling sites at the Poyang Lake Basin.

|  | Sampling sites | Number of individual | Weight (g) |
| --- | --- | --- | --- |
| YR | SW2 | 3 | 3.28 |
|  | SW3 | 1 | 0.35 |
|  | SW4 | 1 | 0.3 |
| CR | SW6 | 0 | 0 |
|  | SW7 | 0 | 0 |
|  | SW8 | 0 | 0 |
| PY | SW9 | 0 | 0 |
|  | SW10 | 2 | 1.4 |
|  | SW17 | 3 | 4.42 |
|  | SW11 | 1 | 0.14 |
|  | SW12 | 2 | 0.0139 |
|  | SW13 | 2 | 0.0148 |
|  | SW14 | 13 | 0.0254 |
|  | SW15 | 0 | 0 |
|  | SW16 | 0 | 0 |
| GJ | SW18 | 0 | 0 |
|  | SW19 | 0 | 0 |
|  | SW20 | 0 | 0 |
|  | SW21 | 0 | 0 |
|  | SW22 | 0 | 0 |
| XH | SW23 | 1 | 0.5 |
|  | SW24 | 0 | 0 |
| FH | SW25 | 0 | 0 |
|  | SW26 | 1 | 4.64 |
| XJ | SW27 | 0 | 0 |
|  | SW28 | 0 | 0 |
| RH | SW29 | 0 | 0 |
|  | SW30 | 0 | 0 |

Number of individual and weight of *Corbicula fluminea* in spring among the different habitats and sampling sites at the Poyang Lake Basin.

|  | Sampling sites | Number of individual | Weight (g) |
| --- | --- | --- | --- |
| YR | SW2-1 | 0 |  |
|  | SW2-2 | 0 |  |
|  | SW2-3 | 0 |  |
|  | SW3-1 | 0 |  |
|  | SW3-2 | 0 |  |
|  | SW3-3 | 0 | 0 |
|  | SW4-1 | 2 | 1.9321 |
|  | SW4-2 | 2 | 1.4676 |
|  | SW4-3 | 0 | 0 |
| CR | SW6-1 | 0 |  |
|  | SW6-2 | 0 |  |
|  | SW6-3 | 0 |  |
|  | SW7-1 | 4 | 1.9401 |
|  | SW7-2 | 9 | 2.6142 |
|  | SW7-3 | 2 | 0.6427 |
|  | SW8-1 | 11 | 1.7662 |
|  | SW8-2 | 30 | 2.0328 |
|  | SW8-3 | 0 | 0 |
| PY | SW9-1 | 5 | 0.3023 |
|  | SW9-2 | 4 | 0.1583 |
|  | SW9-3 | 1 | 0.0391 |
|  | SW10-1 | 0 | 0 |
|  | SW10-2 | 0 | 0 |
|  | SW10-3 | 1 | 0.1368 |
|  | SW17-1 | 3 | 5.7197 |
|  | SW17-2 | 3 | 10.3824 |
|  | SW17-3 | 17 | 20.3921 |
|  | SW11-1 | 3 | 0.2999 |
|  | SW11-2 | 1 | 0.0673 |
|  | SW11-3 | 0 | 0 |
|  | SW12-1 | 0 | 0 |
|  | SW12-2 | 0 | 0 |
|  | SW12-3 | 0 | 0 |
|  | SW13-1 | 0 | 0 |
|  | SW13-2 | 0 |  |
|  | SW13-3 | 1 | 0.6536 |
|  | SW14-1 | 9 | 1.8552 |
|  | SW14-2 | 19 | 4.0941 |
|  | SW14-3 | 23 | 3.3242 |
|  | SW15-1 | 0 | 0 |
|  | SW15-2 | 0 | 0 |
|  | SW15-3 | 0 | 0 |
|  | SW16-1 | 2 | 0.6967 |
|  | SW16-2 | 0 | 0 |
|  | SW16-3 | 0 | 0 |
| GJ | SW18-1 | 0 | 0 |
|  | SW18-2 | 0 | 0 |
|  | SW18-3 | 0 | 0 |
|  | SW19-1 | 0 | 0 |
|  | SW19-2 | 0 | 0 |
|  | SW19-3 | 0 | 0 |
|  | SW20-1 | 0 | 0 |
|  | SW20-2 | 0 | 0 |
|  | SW21-1 | 0 | 0 |
|  | SW21-2 | 0 | 0 |
|  | SW22-1 | 0 | 0 |
|  | SW22-2 | 0 | 0 |
| XH | SW23-1 | 0 | 0 |
|  | SW23-2 | 0 | 0 |
|  | SW24-1 | 0 | 0 |
|  | SW24-2 | 0 | 0 |
| FH | SW25-1 | 0 | 0 |
|  | SW25-2 | 0 | 0 |
|  | SW26-1 | 0 | 0 |
|  | SW26-2 | 0 | 0 |
| XJ | SW27-1 | 0 | 0 |
|  | SW27-2 | 0 | 0 |
|  | SW28-1 | 0 | 0 |
|  | SW28-2 | 0 | 0 |
| RH | SW29-1 | 0 | 0 |
|  | SW29-2 | 0 | 0 |
|  | SW30-1 | 0 | 0 |
|  | SW30-2 | 0 | 0 |

Number of individual and weight of *Corbicula fluminea* in summer among the different habitats and sampling sites at the Poyang Lake Basin.

|  | Sampling sites | Number of individual | Weight (g) |
| --- | --- | --- | --- |
| YR | SW2-1 | 0 | 0 |
|  | SW2-2 | 0 | 0 |
|  | SW2-3 | 0 | 0 |
|  | SW3-1 | 0 | 0 |
|  | SW3-2 | 0 | 0 |
|  | SW3-3 | 0 | 0 |
|  | SW4-1 | 0 | 0 |
|  | SW4-2 | 0 | 0 |
|  | SW4-3 | 0 | 0 |
| CR | SW6-1 | 0 | 0 |
|  | SW6-2 | 0 | 0 |
|  | SW6-3 | 0 | 0 |
|  | SW7-1 | 0 | 0 |
|  | SW7-2 | 0 | 0 |
|  | SW7-3 | 0 | 0 |
|  | SW8-1 | 0 | 0 |
|  | SW8-2 | 23 | 33.0226 |
|  | SW8-3 | 0 | 0 |
| PY | SW9-1 | 6 | 40.5687 |
|  | SW9-2 | 0 | 0 |
|  | SW9-3 | 2 | 11.7299 |
|  | SW10-1 | 0 | 0 |
|  | SW10-2 | 0 | 0 |
|  | SW10-3 | 0 | 0 |
|  | SW17-1 | 1 | 0.8624 |
|  | SW17-2 | 0 | 0 |
|  | SW17-3 | 0 | 0 |
|  | SW11-1 | 2 | 10.1362 |
|  | SW11-2 | 0 | 0 |
|  | SW11-3 | 0 | 0 |
|  | SW12-1 | 0 | 0 |
|  | SW12-2 | 0 | 0 |
|  | SW12-3 | 0 | 0 |
|  | SW13-1 | 0 | 0 |
|  | SW13-2 | 0 | 0 |
|  | SW13-3 | 1 | 0.7197 |
|  | SW14-1 | 0 | 0 |
|  | SW14-2 | 3 | 1.3763 |
|  | SW14-3 | 0 | 0 |
|  | SW15-1 | 0 | 0 |
|  | SW15-2 | 0 | 0 |
|  | SW15-3 | 0 | 0 |
|  | SW16-1 | 1 | 2.4613 |
|  | SW16-2 | 5 | 7.0795 |
|  | SW16-3 | 5 | 9.8436 |
| GJ | SW18-1 | 0 | 0 |
|  | SW18-2 | 0 | 0 |
|  | SW19-1 | 1 | 2.8446 |
|  | SW19-2 | 0 | 0 |
|  | SW20-1 | 0 | 0 |
|  | SW20-2 | 0 | 0 |
|  | SW21-1 | 0 | 0 |
|  | SW21-2 | 0 | 0 |
|  | SW22-1 | 0 | 0 |
|  | SW22-2 | 0 | 0 |
| XH | SW23-1 | 0 | 0 |
|  | SW23-2 | 0 | 0 |
|  | SW24-1 | 0 | 0 |
|  | SW24-2 | 0 | 0 |
| FH | SW25-1 | 0 | 0 |
|  | SW25-2 | 0 | 0 |
|  | SW26-1 | 0 | 0 |
|  | SW26-2 | 1 | 5.4397 |
| XJ | SW27-1 | 0 | 0 |
|  | SW27-2 | 0 | 0 |
|  | SW28-1 | 0 | 0 |
|  | SW28-2 | 0 | 0 |
| RH | SW29-1 | 0 | 0 |
|  | SW29-2 | 0 | 0 |
|  | SW30-1 | 0 | 0 |
|  | SW30-2 | 0 | 0 |
|  | SW30-3 | 0 | 0 |

Number of individual and weight of *Corbicula fluminea* in autumn among the different habitats and sampling sites at the Poyang Lake Basin.

|  | Sampling sites | Number of individual | Weight (g) |
| --- | --- | --- | --- |
| YR | SW2-1 | 0 | 0 |
|  | SW2-2 | 0 | 0 |
|  | SW2-3 | 0 | 0 |
|  | SW3-1 | 0 | 0 |
|  | SW3-2 | 0 | 0 |
|  | SW3-3 | 0 | 0 |
|  | SW4-1 | 0 | 0 |
|  | SW4-2 | 0 | 0 |
|  | SW4-3 | 0 | 0 |
| CR | SW6-1 | 0 | 0 |
|  | SW6-2 | 0 | 0 |
|  | SW6-3 | 0 | 0 |
|  | SW7-1 | 4 | 3.1914 |
|  | SW7-2 | 1 | 0.0426 |
|  | SW7-3 | 0 | 0 |
|  | SW8-1 | 1 | 1.3016 |
|  | SW8-2 | 4 | 5.4146 |
|  | SW8-3 | 11 | 19.0907 |
| PY | SW9-1 | 0 | 0 |
|  | SW9-2 | 0 | 0 |
|  | SW9-3 | 0 | 0 |
|  | SW10-1 | 0 | 0 |
|  | SW10-2 | 0 | 0 |
|  | SW10-3 | 0 | 0 |
|  | SW17-1 | 0 | 0 |
|  | SW17-2 | 0 | 0 |
|  | SW17-3 | 0 | 0 |
|  | SW11-1 | 0 | 0 |
|  | SW11-2 | 2 | 1.4778 |
|  | SW11-3 | 0 | 0 |
|  | SW12-1 | 0 | 0 |
|  | SW12-2 | 1 | 5.4717 |
|  | SW12-3 | 0 | 0 |
|  | SW13-1 | 0 | 0 |
|  | SW13-2 | 0 | 0 |
|  | SW13-3 | 0 | 0 |
|  | SW14-1 | 0 | 0 |
|  | SW14-2 | 2 | 0.9403 |
|  | SW14-3 | 0 | 0 |
|  | SW15-1 | 2 | 0.8248 |
|  | SW15-2 | 0 | 0 |
|  | SW15-3 | 0 | 0 |
|  | SW16-1 | 1 | 0.9833 |
|  | SW16-2 | 2 | 3.7648 |
|  | SW16-3 | 0 | 0 |
| GJ | SW18-1 | 0 | 0 |
|  | SW18-2 | 0 | 0 |
|  | SW19-1 | 0 | 0 |
|  | SW19-2 | 0 | 0 |
|  | SW20-1 | 0 | 0 |
|  | SW20-2 | 0 | 0 |
|  | SW21-1 | 0 | 0 |
|  | SW21-2 | 0 | 0 |
|  | SW22-1 | 0 | 0 |
|  | SW22-2 | 0 | 0 |
| XH | SW23-1 | 0 | 0 |
|  | SW23-2 | 0 | 0 |
|  | SW24-1 | 0 | 0 |
|  | SW24-2 | 0 | 0 |
| FH | SW25-1 | 0 | 0 |
|  | SW25-2 | 0 | 0 |
|  | SW26-1 | 0 | 0 |
|  | SW26-2 | 0 | 0 |
| XJ | SW27-1 | 5 | 14.3713 |
|  | SW27-2 | 3 | 11.8089 |
|  | SW28-1 | 0 | 0 |
|  | SW28-2 | 1 | 2.8476 |
| RH | SW29-1 | 0 | 0 |
|  | SW29-2 | 0 | 0 |
|  | SW30-1 | 0 | 0 |
|  | SW30-2 | 0 | 0 |
|  | SW30-3 | 0 | 0 |
